# Editorial: Rectus Diastasis

**DOI:** 10.3389/jaws.2025.14296

**Published:** 2025-03-24

**Authors:** Manuel López Cano

**Affiliations:** Abdominal Wall Sugary Unit, Department of General Surgery, Hospital Universitari Vall d'Hebron, Universitat Autónoma de Barcelona, Barcelona, Spain

**Keywords:** rectus diastasis, diastasis recti, abdominal wall surgery, hernia, linea alba

Rectus diastasis (RD) is a hot topic in surgical practice related to abdominal wall surgery. RD describes the separation of the rectus abdominis muscles and is characterised by widening of the linea alba. This causes the midline to “bulge” when intra-abdominal pressure is increased. RD is not a hernia because it does not have a true fascial defect although it can be associated with primary midline hernias. It is a condition mostly seen in women after pregnancy and, to a lesser extent, in obese men. Open repair techniques have been described although nowadays the development of minimally invasive approaches for treating RD, has led to a consideration on the role that these novel techniques may play. Despite the increase in ways of approaching RD there is a lack of consensus on the definition, diagnosis and therapeutic management of RD.

Some of these controversial aspects are discussed in this special issue. Thus, it describes the prepartum anatomy of the abdominal wall in a cohort of nulliparous women, for use as a reference for management of patients with postpartum abdominal wall insufficiency (Woxnerud et al.). Similarly, Ngo et al. provide additional features concerning the type of bulging and the width of divarication. In relation to the diagnosis and symptomatology of RD the study of van Wingerden et al. provides an inventory of the incidence of RD in subjects with chronic back and pelvic pain and Bixo et al. offer us a study aimed to understand the correlation between the post-partum inter-recti distance and functional impairments associated with core instability. Finally, regarding the treatment Katawazai et al. evaluates the impact of the minimal incision repair of rectus abdominis diastasis (MIRRAD) procedure on physical activity, muscle strength, quality of life, and overall satisfaction in women with postpartum and Mandujano et al. show us a valuable algorithmic approach for minimally invasive surgery for symptomatic ventral hernias with diastasis of the rectus abdominis muscle.

The increase in knowledge about RD and how to apply it at general and specific levels may lead to a greater increase in the cost-effectiveness of this process, a reduction in morbidity, and better health-related quality-of life of our patients. Although this special issue does not cover all the aspects that can be considered in the handling/treatment of RD we hope it will be helpful to interested readers and help improve the management of this controversial entity.

